# Cryopreservation and Rapid Recovery of Differentiated Intestinal Epithelial Barrier Cells at Complex Transwell Interfaces Is Enabled by Chemically Induced Ice Nucleation

**DOI:** 10.1021/acsami.4c03931

**Published:** 2024-04-26

**Authors:** Akalabya Bissoyi, Yanan Gao, Ruben M. F. Tomás, Nina L. H. Kinney, Thomas F. Whale, Qiongyu Guo, Matthew I. Gibson

**Affiliations:** † Department of Chemistry, University of Warwick, Coventry CV4 7AL, U.K.; ‡ Division of Biomedical Sciences, Warwick Medical School, University of Warwick, Coventry CV4 7AL, U.K.; § Department of Chemistry, University of Manchester, Oxford Road, Manchester M13 9PL, U.K.; ∥ Manchester Institute of Biotechnology, University of Manchester, 131 Princess Street, Manchester M1 7DN, U.K.; ⊥ Cryologyx Ltd, Venture Centre, University of Warwick Science Park, Coventry CV4 7EZ, U.K.; # Department of Biomedical Engineering, Southern University of Science and Technology, Shenzhen, Guangdong 518055, China; ¶ School of Earth and Environment, University of Leeds, Leeds LS2 9JT, U.K.; ∇ Royal Botanic Gardens Kew, Ardingly, West Sussex RH17 6TN, U.K.

**Keywords:** cryopreservation, nucleation, Caco-2 cells, toxicology, ice

## Abstract

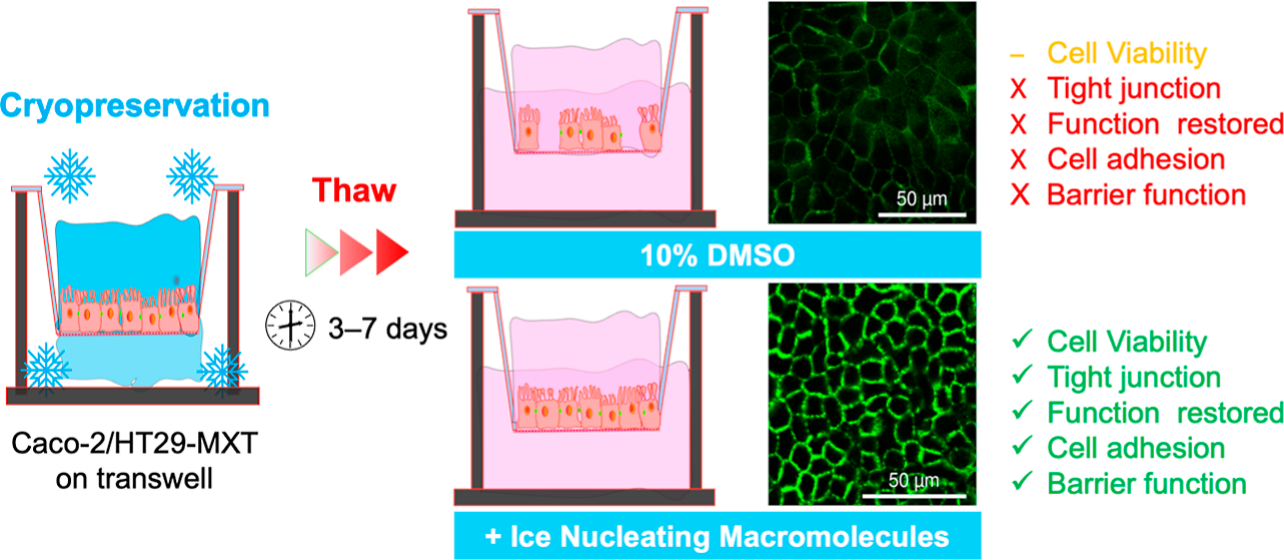

Cell-based models, such as organ-on-chips, can replace and inform in vivo (animal) studies for drug discovery, toxicology, and biomedical science, but most cannot be banked “ready to use” as they do not survive conventional cryopreservation with DMSO alone. Here, we demonstrate how macromolecular ice nucleators enable the successful cryopreservation of epithelial intestinal models supported upon the interface of transwells, allowing recovery of function in just 7 days post-thaw directly from the freezer, compared to 21 days from conventional suspension cryopreservation. Caco-2 cells and Caco-2/HT29-MTX cocultures are cryopreserved on transwell inserts, with chemically induced ice nucleation at warmer temperatures resulting in increased cell viability but crucially retaining the complex cellular adhesion on the transwell insert interfaces, which other cryoprotectants do not. Trans-epithelial electrical resistance measurements, confocal microscopy, histology, and whole-cell proteomics demonstrated the rapid recovery of differentiated cell function, including the formation of tight junctions. Lucifer yellow permeability assays confirmed that the barrier functions of the cells were intact. This work will help solve the long-standing problem of transwell tissue barrier model storage, facilitating access to advanced predictive cellular models. This is underpinned by precise control of the nucleation temperature, addressing a crucial biophysical mode of damage.

## Introduction

Preclinical drug discovery fundamentally relies on cell-based models to enable the screening of new leads and identification of pathways and modes of action. Over 92% of drugs fail to translate from animal testing to human treatments, primarily due to unexpected toxicity or lack of efficacy.^1^ However, innovative tools such as organs-on-chips and three-dimensional cell culture, which better recapitulate human physiology, can identify 87% of drugs that cause human liver damage, representing more accurate safety and efficacy testing.^2−8^ Hence, cell-based tools accelerate drug development and reduce the costs and risks associated with animal and human clinical trial failure rates.

Complex cell models used in absorption, distribution, metabolism and excretion (ADME) studies require advanced infrastructure and expertise to prepare and validate for bespoke use, often taking several weeks.^9,10^ For example, enterocytes form epithelial layers that mimic intestinal barriers for drug absorption testing, but successful testing depends on careful and prolonged culturing to display critical differentiated features, such as tight junctions and passive/active transporters.^11^ The complexity of model generation, combined with storage and logistical challenges, slows access and adoption of the model within testing laboratories. An ideal solution would be to prepare these models at a centralized site and cryopreserve them for mass storage and ease of transport to end users.

Caco-2 cells (human colorectal epithelial adenocarcinoma) and cocultures of Caco-2 and HT29-MTX (methotrexate-resistant human colorectal epithelial adenocarcinoma) cells are cultured on porous transwell inserts to replicate the intestinal barrier. This setup is widely utilized for monitoring drug permeability through the epithelial barrier, facilitating accurate studies of both passive and active drug absorption dynamics.^12−16^ However, Caco-2 cells are currently stored cryopreserved in a suspension within a cryovial. Thus, to establish this model, thawed cells must undergo an expansion process for approximately 16 days until sufficient cell quantities are obtained and cultured on transwells for over 20 days to express the necessary enterocyte and colonocyte phenotypes essential for drug absorption testing. This process presents a significant logistical challenge as drug absorption studies can require up to 40 days to complete. Consequently, any disruptions due to contamination can lead to substantial delays, making the continuous maintenance of cultures both time- and resource-intensive. The cryopreservation of cell monolayers on transwells at the point of differentiation would significantly simplify these processes, offering a ready to use standardized intestinal model straight from the freezer to advance the use and access of nonanimal models.

Cryopreservation enables long-term banking and distribution of cells and is commonly achieved using organic solvent cryoprotectants, with 5 to 10% DMSO being the most widely used cryoprotectant for nucleated mammalian cells. This enables cryopreservation of many cells in suspension, but cell monolayers pose significantly more challenges, with recoveries in the range of 10–30% compared to above 70% in suspension.^17^ Macromolecular cryoprotectants, based on ice-binding proteins or synthetic polymers (especially polyampholytes),^17^ have been shown to dramatically improve monolayer storage of cells, especially in larger microwell formats (e.g., 12- and 24-well plates). Gibson et al. have reported near 100% recovery of various cell lines including primary cell monolayers.^18^ Cryopreserving cells in smaller microwells, such as 96-well plates, are especially challenging due to a delay in ice nucleation of smaller volumes (supercooling), whereby ice formation can occur at −15 °C.^18,19^ Extracellular ice formation is required to increase osmotic gradients and promote cellular dehydration, so supercooling can lead to lethal intracellular ice formation.^20^ There is also high well-to-well variability due to the stochastic nature of ice nucleation, leading to a range of freezing values.

Chemically induced ice nucleation can control the temperature of ice formation to minimize supercooling and its deleterious effects. For example, the ice nucleator feldspar^19^ (a mineral) has been shown to improve the post-thaw outcomes of cell monolayers cryopreserved in 96-well microplates. However, specialist devices are required to remove the insoluble mineral from the cells, which are incompatible with complex cellular models and transwell inserts. There are few reports of chemically defined ice nucleators, with some dispersible carbon nanomaterials^21^ and dense polymer brushes^22^ showing moderate ice nucleation activity. Soluble polysaccharides extracted from plant pollen have displayed remarkable ice nucleation properties.^23,24^ By raising the ice nucleation temperature to as high as −4 °C, cryo-injury is minimized during freezing and thawing to enhance cell recovery and preserve function.^25^ The exact role and evolutionary/incidental origin of these nucleators is not yet understood,^26^ but their solubility enables easy deployment and post-thaw wash out. Based on the limited studies available, the inclusion of ice nucleators from pollen has shown potential to retain cell integrity and viability during freezing and thawing procedures.^25,27^

Here, we present the successful cryopreservation and recovery of differentiated Caco-2 (and coculture) epithelial barriers directly on transwell interfaces, reducing model preparation time from approximately 40 days to less than 7 days. Ice-nucleating macromolecules were utilized to elevate the temperature of ice formation and effectively prevent supercooling, which would otherwise result in cell death and detachment. A panel of functional assays, including electrical resistance, dye permeation, whole-cell proteomics, and histology, were employed to validate the protective effect of chemically induced ice nucleation, demonstrating that the cells remain both viable and functional. This marks a paradigm shift in cryopreservation capabilities to models previously impossible to cryopreserve, potentially broadening the access of complex animal-free models by alleviating logistical burdens and simplifying the ease of use.

## Experimental Section

For additional experimental methods, see the Supporting Information.

### Materials

Minimum essential medium Eagle medium (MEM, M4655), non-US origin fetal bovine serum (FBS, F7524), MEM nonessential amino acid solution 100× (M7145), Dulbecco’s phosphate-buffered saline (DPBS, D8537), dimethyl sulfoxide (Hybri-max, sterile-filtered) (D2650), Triton X-100 (X100), 0.4% trypan blue (T8154), RNase A from bovine pancreas, Corning CoolCell LX cell freezing vial container (CLS432001), and CorningXT CoolSink96F thermoconductive plate (CLS432070) were purchased from Merck (Gillingham, UK). Live/dead viability assay kit, ethidium homodimer-1 (EI) and calcein-AM (L3224), Corning 96-well white polystyrene microplates (10022561), trypsin (0.25%) and EDTA phenol red (500 mL) (25200072), Invitrogen ActinGreen 488 ReadyProbe reagent (R37110), antibiotic–antimycotic solution 100× (15240062), and cryovials were purchased from Thermo Fisher (Loughborough, UK). The Caco-2 cells were purchased from ATCC, USA, and the HT29-MTX cell line at passage 4 was kindly provided by Prof Sebastien Perrier, Warwick University, UK. MycoAlert Mycoplasma Detection Kit (LT07–703) was purchased from Lonza (Basel, Switzerland). Spectrum Laboratories Spectra/Por 2 12–14 kDa MWCO (15390762) and Corning 96-Well Clear Ultra Low Attachment Microplates (10023683) were purchased from Fisher Scientific (Loughborough, UK). A WST-1 proliferation reagent and protease and phosphate inhibitor cocktail (ab201119) were purchased from Abcam (Cambridge, UK). Hoechst 33342 was purchased from Life Technologies (CA, USA). Purified primary antibody Mouse Anti-Human zonula occludens-1 (ZO-1) and secondary antibody FITC Goat Anti-Mouse Ig were purchased from BD Pharmingen, UK. Transwell 24-well plate with 0.33 cm^2^ permeable polycarbonate membrane inserts were purchased from Corning (Fisher Scientific, UK). European hornbeam (*Carpinus betulus*) pollen was purchased from Pharmallerga. Lucifer Yellow CH dipotassium salt was purchased from Sigma, UK.

### Cell Culture

Caco-2 and HT29-MTX were cultured in Dulbecco’s modified Eagle’s medium (DMEM) supplemented with 10% FBS, 2 mM l-glutamine, and 1% penicillin–streptomycin (Invitrogen, USA) in a humidified incubator at 37 °C and 5% CO_2_. The cells were routinely passaged in a T175 flask every 3–4 days, before reaching 70–80% confluency. Cells were dissociated by using a balanced salt solution containing trypsin (0.25%) and EDTA (1 mM). Mycoplasma contamination was tested routinely with a MycoAlert Mycoplasma Detection Kit 150.

### Preparing Cryoprotectant Formulation

European hornbeam pollen (0.8 g) was suspended in 10 mL of Milli-Q water at 4 °C overnight and sterile filtered using a 0.22 μm filter. The solution was mixed 1:1 with either MEM supplemented with 20% DMSO, 20% FBS, and 1 × 10^6^ Caco-2 cells for the apical portion or MEM supplemented with 20% DMSO and 20% FBS for the basal portion. The final DMSO concentration was 10% DMSO. Figures S1–S5 in the Supporting Information Section provide a detailed description of the ice nucleator solution’s characteristics. The resulting solution is called Ice nucleator (IN) throughout the manuscript.

### Cryopreservation of Caco-2 Cells in Transwell Inserts

Corning membrane transwell inserts (0.33 cm^2^) were placed in a 24-well plate. To the apical chamber, 200 μL of collagen rat tail type I (0.15 mg/mL) was added, UV sterilized for 30 min, and incubated overnight in a sterile biosafety cabinet at 37 °C with 5% CO_2_. The collagen was removed, and the insets were washed once with DPBS. Caco-2 cells were seeded on the collagen-coated transwell inserts at a density of 3 × 10^5^ Caco-2 cells/mL (200 μL). The cells were incubated for 4 h, to allow cell attachment, and the medium in the upper compartment (apical portion) was replaced with 200 μL of fresh medium, and 750 μL was added to the lower compartment (basal portion). Caco-2 cells were incubated at 37 °C and 5% CO_2_ for 14 or 21 days, with medium changes every 2 days. The medium was removed and 50 μL of the apical cryoprotectant solution (containing Caco-2 cells) and 50 μL of the basal cryoprotectant solution (no cells) was added. Following 10 min of incubation at room temperature, the 24-well plates were placed on a CorningXT CoolSink96F thermoconductive plate and frozen in a–80 °C freezer overnight.^28^ Cells were also cryopreserved with solutions without the addition of ice nucleating molecules from pollen, for comparison. Cells were thawed by adding 250 μL of prewarmed cell culture medium on the apical side of the transwell and 750 μL on the basolateral side. Cell culture medium was replaced with fresh medium after 2 h of incubation at 37 °C and 5% CO_2_.

### Cryopreservation of Caco-2/HT29-MTX Coculture in Transwell Inserts

Corning membrane transwell inserts (0.33 cm^2^) were placed in a 24-well plate. To the apical chamber, 200 μL of collagen rat tail type I (0.15 mg/mL) was added, UV sterilized for 30 min and incubated overnight in a sterile biosafety cabinet at 37 °C with 5% CO_2_. The collagen was removed, and the insets were washed once with DPBS. A mixture of Caco-2 cells and HT29-MTX cells was seeded at a density of 3 × 10^5^ cells/mL and 1 × 10^5^ cells/mL, respectively. The cells were incubated for 4 h, to allow cell attachment, and the medium in the upper compartment (apical portion) was replaced with 200 μL of fresh medium and 750 μL was added to the lower compartment (basal portion). Caco-2 cells were incubated at 37 °C and 5% CO_2_ for 14 or 21 days, with medium changes every 2 days. The medium was removed and 50 μL of the apical cryoprotectant solution (containing Caco-2 cells) and 50 μL of the basal cryoprotectant solution (no cells) was added. Following 10 min of incubation at room temperature, the 24-well plates were placed on a CorningXT CoolSink96F thermoconductive plate and frozen in a −80 °C freezer overnight. Cells were also cryopreserved with solutions without the addition of ice nucleating molecules from pollen for comparison. Cells were thawed by adding 250 μL of prewarmed cell culture medium on the apical side of the transwell and 750 μL on the basolateral side. Cell culture medium was replaced with fresh medium after 2 h of incubation at 37 °C and 5% CO_2_.

### Cytoskeletal and ZO-1 Staining

The nucleus, actin, and ZO-1 proteins of Caco-2 cells were stained 3, 7, 14, and 21 days after culture to monitor structural changes and 1 and 3 days after freeze/thaw with 10% DMSO and 10% DMSO plus intracellular ice nucleators. The nucleus, actin, and ZO-1 proteins of Caco-2/HT29-MTX cocultures were also stained 21 days after culturing and 24 h post-thaw after freeze/thaw with 10% DMSO and 10% DMSO plus intracellular ice nucleators. To complete this, cells were fixed with 1% paraformaldehyde for 10 min and washed with DPBS. Cells were blocked in the apical compartment with 1% BSA and 50% goat serum for 30 min. After three washes, the cells were incubated with a primary antibody Mouse Anti-Human ZO-1 (250 μg/mL) at 1:100 dilution in 0.1% BSA overnight. Cells were washed three times with DPBS before being incubated with 10 μg of FITC Goat Anti-Mouse IgG secondary antibodies. After three washes with DPBS, Hoechst 33342 was added for 5 min. After additional washes, the polycarbonate membrane was cut out using a scalpel and mounted on a glass slide with a ProLong Gold Antifade. Cells were finally observed using an Olympus FV 3000 confocal microscope using 405, 488, and 561 nm using dry 20× objective, and images were analyzed using fiji 1.48 and prepared using OMERO.web 5.22.1(Warwick university imaging facility).

### Trans-Epithelial Electrical Resistance (TEER) Measurement

For TEER measurement, an EVOM2 (World Precision Instruments, UK) was fitted with sterilized STX2 electrode probes. TEER values were recorded for Caco-2 and Caco-2/HT29-MTX cocultures after 3–21 days of culture and 1–4 days after freeze/thaw with 10% DMSO with and without ice nucleators. Transport buffer (TB) was prepared containing 25 mM glucose and 10 mM HEPES. The pH was adjusted to 7.4 by using 1 M sodium hydroxide solution. The TB was placed in an incubator set at 37 °C with 5% CO_2_ for 2 h. TB (250 μL)was added to the apical portion, and 750 μL volume of TB was added to the basolateral portion of the transwells containing cells. The electrode probes were placed in the TB, and resistance was measured in ohms (Ω) and multiplied by the surface area of the transwell. Data were analyzed using GraphPad Prism software.

### Paracellular Permeability Test

Lucifer yellow (LY) permeability tests were carried out for Caco-2, HT29-MTX, and Caco-2 and HT29-MTX cococultures after 1–14 days of culture and 1, 2, and 7 days after freeze/thaw using 10% DMSO and 10% DMSO supplemented with ice nucleators. Cells were washed with DPBS once and incubated with 200 μL volume of LY (1 mg/mL) on the apical side and 900 μL of HBSS on the basolateral side of the membrane for 1 h at 37 °C. The fluorescence intensity in the apical and basal compartments was measured using a spectrofluorometer (PerkinElmer, USA) at an excitation wavelength of 430 nm and an emission wavelength of 540 nm.

### Alkaline Phosphatase (ALP) Assay

The activity of ALP was assessed in cells cultured for 21 days in both systems. We utilized an ALP colorimetric assay kit (ab83369, Abcam, Cambridge, UK) according to the manufacturer’s protocol. In brief, we extracted membranes and inserts from the transwell chambers. After rinsing the cells with HBSS at 37 °C, trypsin/EDTA was applied, and the cells were incubated for 5–7 min. Following this, we collected the cell suspension, centrifuged it at 300*g* for 5 min at 4 °C, and resuspended the cell pellet in 200 μL of ALP assay buffer. Subsequently, we centrifuged at maximum speed (16,000 rpm) for 5 min at 4 °C. The supernatant (sample) was collected and pipetted into the wells of a 96-well plate. A reaction buffer (50 μL/well) containing a *p*-nitrophenyl phosphate solution (5 mM) was added. After incubating the plate in the dark for 60 min at 25 °C, we introduced 20 μL of stop solution to each well and gently shook the mixture. The absorbance was promptly read at 405 nm using a microplate reader.

### Proteomics

Sample Lysis. Cells were washed with ice-cold phosphate-buffered saline (PBS) and lysed using RIPA lysis buffer (100 μL per 10^6^ cells) supplemented with a protease inhibitor cocktail with 1:9 dilution. Cells were homogenized using a cell scraper, and samples were transferred into microtubes and incubated on ice for 30 min, gently vortexing periodically. The samples were sonicated with the Bioruptor Plus (UCD-300) for 2 min in an ice bath for protein extraction. The lysate was centrifuged at 12,000*g* for 20 min at 4 °C, and the supernatant was transferred to a new chilled microcentrifuge tube. Protein content was quantified using NanoDrop 2000/2000c (Thermo Fisher Scientific, USA). Buffer Exchange. The protein samples were added to filter units, and 400 μL of 50 mM ammonium bicarbonate (ABC) was added. The samples were centrifuged at 8000*g* for 20 min, and this process was repeated three times. Reduction and Alkylation. To the resulting solution was added 400 μL of tris(2-carboxyethyl)phosphine (TCEP) (10 mM), chloroacetic acid (CAA) (10 mM), and ABC (50 mM) for 30 min at room temperature. The solution was then removed by centrifugation at 8000*g* for 20 min, and 400 μL of 50 mM ABC was used to wash the filter and centrifuged at 8000*g* for 20 min. Protein Digestion. The supernatant was replaced with 400 μL of ABC (50 mM) supplemented with 2 μg of trypsin per 100 μg of protein, and the mixture was incubated at 37 °C overnight. Peptide Elution. The filters were transferred to new collection tubes and centrifuged at 8000*g* for 20 min. Water (400 μL) was added to the filter tubes and centrifuged at 8000*g* for 20 min. All solutions were centrifuged at 1000*g* and 60 °C for 1.5 h to evaporate water. Final protein solution (50 μL) was added to the tubes, and the samples were ready for mass spectrometry analysis using an ion mobility Q-ToF mass spectrometer with nanoElute UPLC (Bruker). Data processing and analysis were carried out by Scaffold5.3.0 and Perseus software.

### Hematoxylin and Eosin (H&E) Staining

Caco-2 on transwells were cultured for 14 days as a control group, and the samples were cryopreserved with 10% DMSO with or without ice nucleators and thawed as experimental groups. The samples were collected from transwells and fixed in 4% paraformaldehyde at 4 °C for 24 h. They were embedded in the O.C.T. Compound Mounting Medium for Cryotomy (VWR Q Path, 00411242) in cryomolds (Agar Scientific Ltd., UK, AGG4581), placed on dry ice for 30 min, and stored at −80 °C before use. A cryostat (Epredia CryoStar NX50) was used for the cryosection. Optimal cutting temperature (OCT) compound was placed on the tissue holder of the cryostat at room temperature and then the sample was transferred on it. The mounted holder was placed at −35 °C stage for 20 min for precooling in the cryostat. Each section (10 μm thick) was cut with a motor-driven microtome using a blade (Epredia Ultra Disposable Microtome Blades, MX35,3053835). Sections were unfolded with a brush and transferred on glass slides (VWR International bvba, 631-0107) and left at room temperature to allow samples to stick on slides. The cryosections were washed with deionized water, following the protocol of the H&E staining kit (Generon Ltd., HAE-2). Samples were immersed in fresh hematoxylin solution for 1 min and washed in water twice to remove excess staining solution. Samples were then differentiated in the blueing solution for 30 s and rinsed twice with water. The slides were then dried and placed in eosin for 2 min. The slides were rinsed gently to remove excess eosin. Sections were dehydrated with 100% ethanol for 30 s, then placed in xylene for 30 s. A drop of mounting medium (Sigma-Aldrich, 06522) was placed on the slides with H&E staining sections and covered with a coverslip. The prepared slides were imaged using a light microscope.

### Statistical Analysis

Data were analyzed using GraphPad Prism software (GraphPad Software, USA). The results are presented as the mean ± standard deviation (SD). The significance of the differences between the groups was determined using a one-way ANOVA followed by Tukey’s posthoc test. A *p*-value of <0.05 was considered statistically significant.

## Results and Discussion

**Figure 1:**
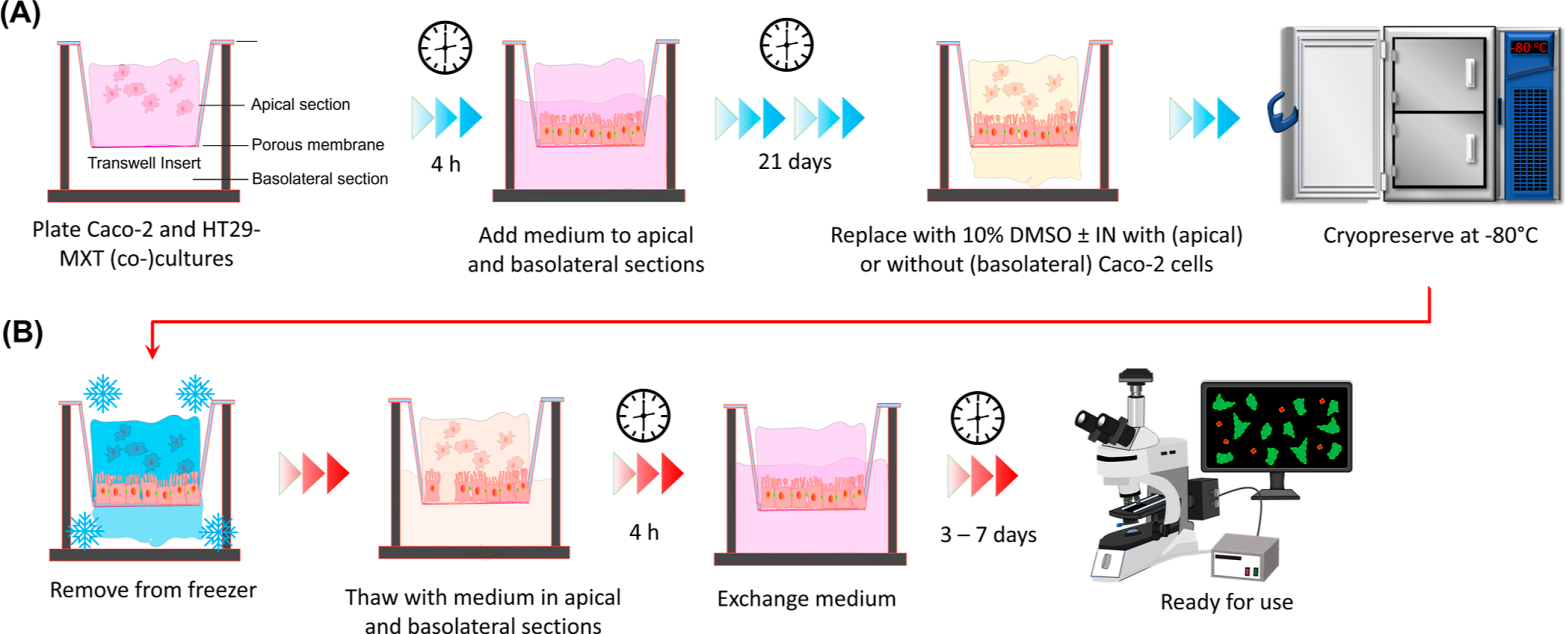
Experimental Overview. (A) Cryopreservation and (B) thawing processes for Caco-2, HT29-MXT, and Caco-2/HT29-MXT coculture on transwells.

Caco-2 (and other epithelial) cells are not intrinsically challenging to cryopreserve in suspension, using 10% DMSO, or as 2-D monolayers in conventional 24-microwell plates when using polyampholytes.^29^ However, the functional usage of Caco-2 cells in drug absorption studies requires culturing on transwells to establish an epithelial barrier consisting of differentiated cells. Among multiple features, the differentiated epithelial barrier forms tight junctions, which are not possible to preserve with existing cryopreservation technology. Loss of the epithelial layer is characterized by cell detachment, loss of tight junctions, reduction in viability, and induction of programed cell death pathways. The integrity of this barrier is critical to the success of permeability studies, making this model one of the most difficult cryopreservation challenges. DMSO alone cannot preserve these critical structures. Delayed ice nucleation, often encountered in 2-D cell monolayer cryopreservation in 96-well plates, promotes disruption of cell–cell and cell–substrate adhesion due to severe temperature fluctuations at local scale.^19^ When induced nucleation is used during the freezing process, extracellular ice forms at warm temperatures, increasing the effective osmotic pressure (as ice forms a pure phase) and aids cellular dehydration and reduces intracellular ice formation. Hence, without nucleation, supercooling can lead to intracellular ice formation and cell death.^27,30,31^ Thus, we hypothesized that the use of a soluble ice nucleator would enable the cryopreservation of epithelial barriers, maintaining the necessary differentiated enterocytic features. To investigate this, Caco-2 and HT29-MTX cells were (co)cultured on transwell inserts for 21 days to enable differentiation (Figure S6), and the confluent epithelial layer was cryopreserved with 10% DMSO supplemented with ice nucleator (+IN) or without ice nucleator (-IN) (the soluble polysaccharide ice nucleator extracted from pollen), Figure 1. This easily removable, soluble ice nucleator has been previously used to aid the cryopreservation of other complex (3-D spheroid) models, by increasing the nucleation temperature from as low as −20 °C (supercooled) to −7 °C.^27,32^Figure S3 shows the ice nucleation temperature curve for the transwell system with or without IN. Post-thaw, the cells were thawed with warm cell culture medium, and a minimum recovery period of 24 h was allowed to remove false positives associated with short post-thaw times.^33^ By preserving differentiated Caco-2 and HT29-MTX phenotypes, this study aimed to reduce the time taken to produce drug absorption models from 21 days of culturing (from an already established culture) to 3–7 days of post-thaw recovery time.

**Figure 2:**
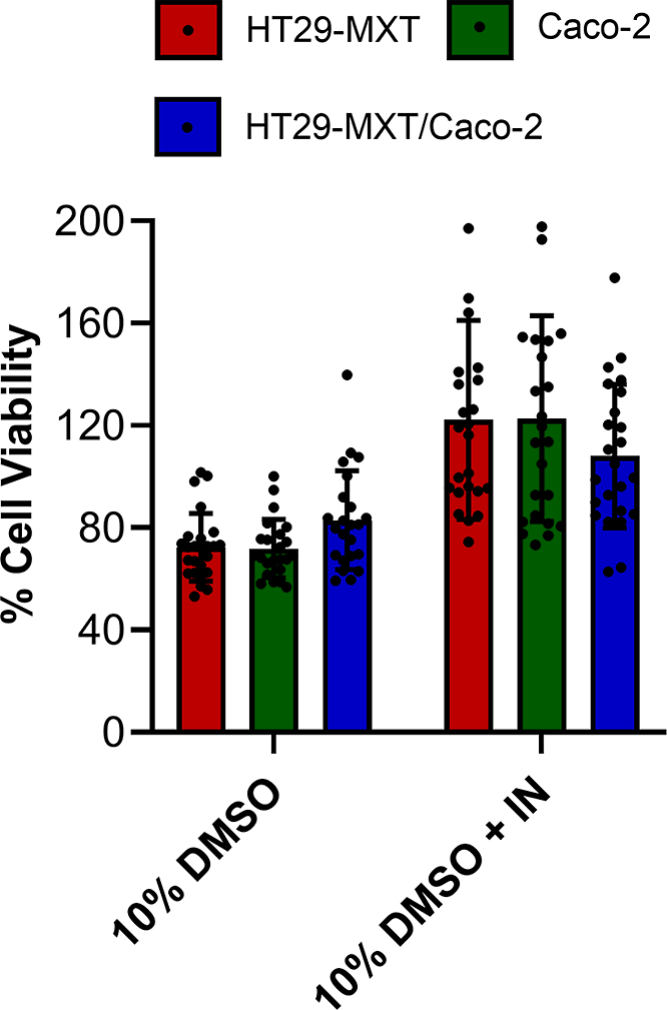
Post-thaw cell viability. Caco-2, HT29-MTX, and Caco-2/HT29-MTX cells were cultured for 21 days and cryopreserved with either 10% DMSO or 10% DMSO plus IN. Cell viability was determined 24 h post-thaw with a WST-8 assay ±SD (5 biological and 5 technical repeats).

Post-thaw cell viability was determined by the WST-1 (metabolic) assay; the results of this are shown in Figure 2. Compared to the nonfrozen control, 75% viability was obtained by cryopreserving the differentiated epithelial layers with 10% DMSO alone, i.e., without chemically induced ice nucleation. In contrast, soluble ice nucleator supplementation within the cryoprotectant medium increased post-thaw cell viability by 40%, reaching 115%, highlighting the remarkable impact of decreasing the extent of supercooling on cryopreservation outcomes. [Note, >100% viability is possible due to the 24 h post-thaw culture where cell number can increase].

**Figure 3:**
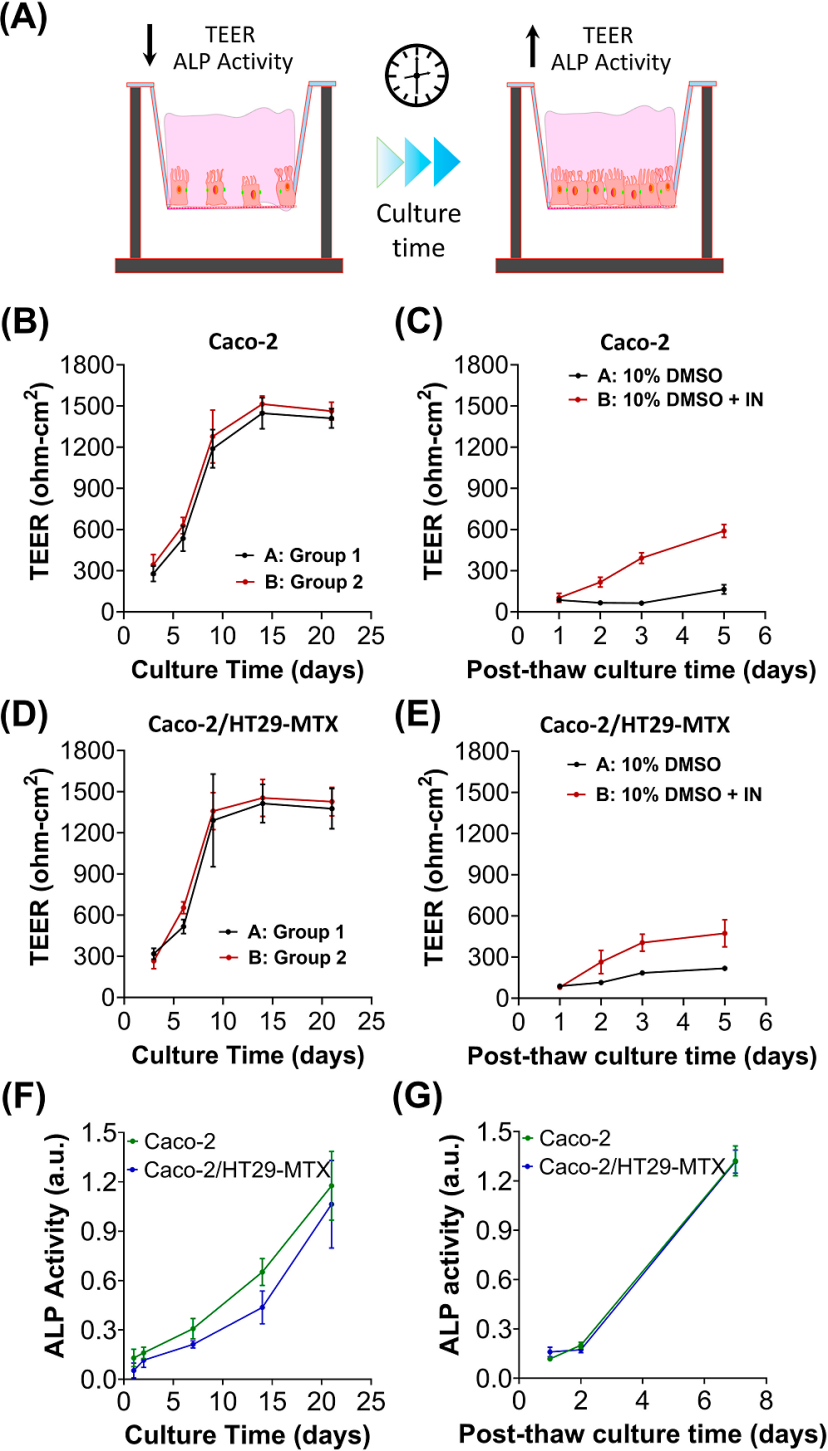
Post-thaw TEER and ALP measurements. (A) Schematic illustrating that TEER and ALP activity increase with culture time once cells become confluent and differentiated. TEER measurements of Caco-2 (B) before and (C) after freeze–thaw with 10% DMSO or 10% DMSO plus IN ± SD (3 biological and 3 technical repeats). TEER measurements of Caco-2/HT-29 cocultures (D) before and (E) after freeze/thaw with 10% DMSO or 10% DMSO plus IN ± SD (3 biological and 3 technical repeats). ALP activity of Caco-2 and Caco-2/HT-29 cocultures (F) before and (G) after freeze/thaw with 10% DMSO plus IN ± SD (3 biological and 3 technical repeats). Group 1 and Group 2 refer to the samples that would be treated with 10% DMSO and 10% DMSO + IN, respectively.

Following confirmation that induced ice nucleation increases the percentage of viable cells recovered post-thaw, the preservation and/or recovery of barrier integrity was assessed by TEER, which is a nondestructive technique. By continuously measuring TEER, the differentiation of Caco-2 and HT29-MTX (co)cultures before cryopreservation was monitored (Figure 3B/D) and compared with post-thaw as an indicator of barrier integrity, Figure 3C/E. The TEER values increased from 300 to 1500 Ω Cm^2^ over the course of 15 days, clearly showing the extended time required for the monolayer to establish. It should be noted that the reported TEER values for these can show a relatively large range.^34^ After 21 days of culture, the epithelial barriers were cryopreserved, as described above, in 10% DMSO with or without induced ice nucleation, and TEER values were recorded 1–5 days post-thaw, Figure 3C. An initial drop in TEER was observed (to ∼100 Ω cm^2^) regardless of ice nucleation temperature, indicating an initial loss of barrier integrity. Cell loss was minimized during cryopreservation, as indicated by cell viability measurements, suggesting that the quantity of cells was not to blame for the reduction in TEER values. Further studies were carried out to probe barrier integrity later in this manuscript. However, the beneficial effects of cryopreserving with an ice nucleator were clear over the 5 day post-thaw recovery period, with TEER values increasing to 600 Ω cm^2^. In contrast, without controlled nucleation, minimal increases in TEER were observed post-thaw, over time, suggesting that extensive supercooling irreversibly harms the epithelial barrier function. The observed TEER value was lower than the fresh monolayer but was still within the range utilized for drug absorption testing.^35−41^

Caco-2 differentiation results in a highly functionalized epithelial barrier with morphological and biochemical similarities to intercellular junctions, well-defined brush borders (microvilli structure), and expression of absorptive transporters.^42^ ALP activity, a brush border-localized hydrolase, was monitored as a marker of enterocytic differentiation during Caco-2 and Caco-2/HT29-MTX (co)culturing and post-thaw, following cryopreservation with 10% DMSO and the ice nucleator, Figure 3G. ALP activity gradually increased from 0.1 to 1.2 over 21 days of culture, Figure 3F. Accumulation of ALP within differentiated Caco-2 cells is expected due an increase in ALP synthesis rates and low turnover rates.^43^ Post-thaw, an initial drop in ALP was observed, but the activity was completely restored within 7 days, suggesting that the cryopreservation of Caco-2 epithelial layers with 10% DMSO and the soluble ice nucleator does not adversely affect differentiated biochemical functions. ALP deletion has been shown to downregulate tight junction adhesion proteins that promote improved epithelial barrier function, including ZO-1, ZO-2, and occludin,^44^ so restoration of ALP activity post-thaw is critical to retain tight junctions.

**Figure 4:**
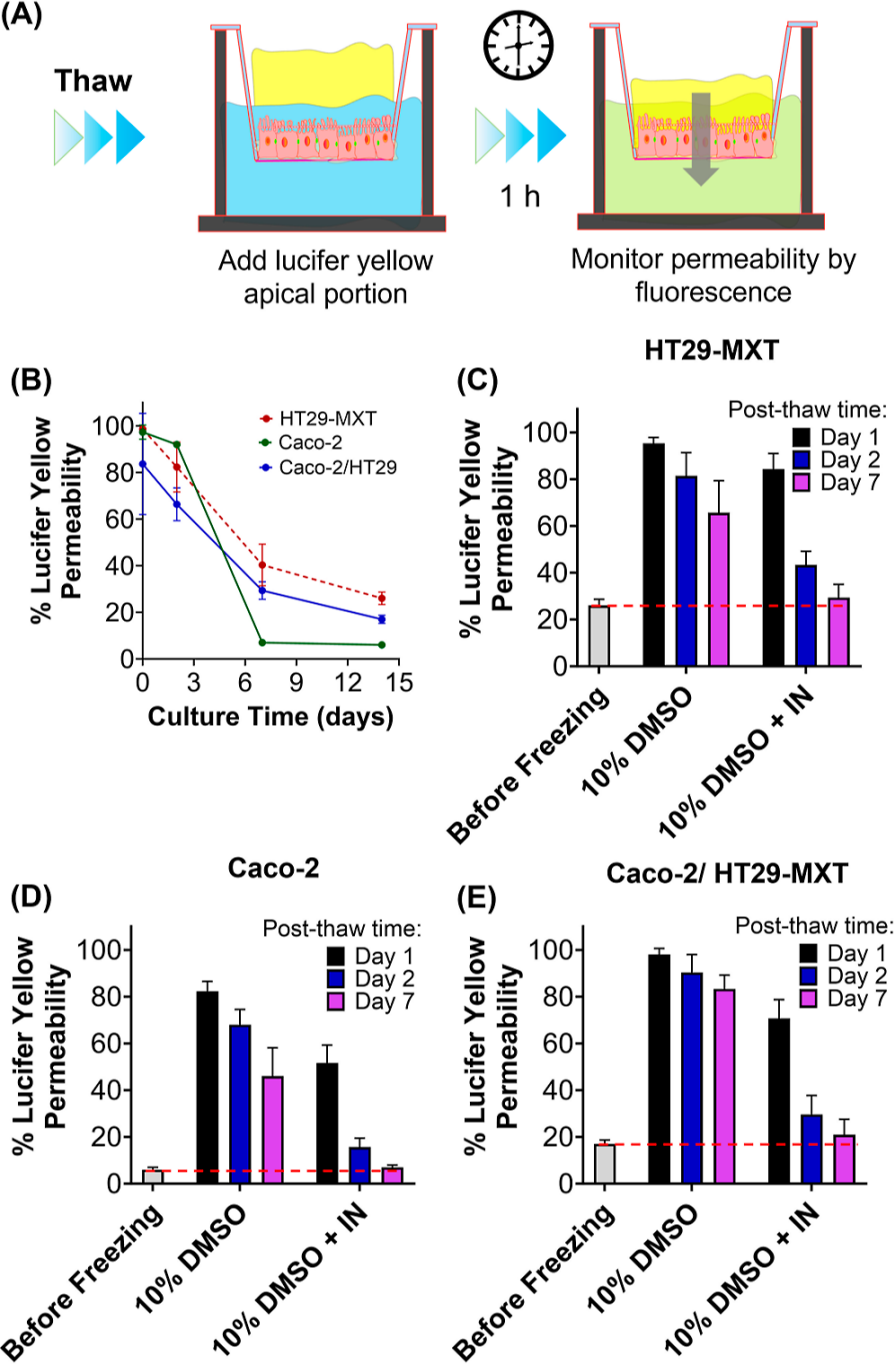
LY permeability test. (A) Schematic of the LY permeability assay. A LY permeability functionality assay was used to monitor tight junctions (B) before freezing and after freeze/thaw using 10% DMSO and 10% DMSO plus IN in (C) HT29-MXT, (D) Caco-2, and (E) Caco-2/HT29-MXT cocultures. Percentage LY permeability was reported ±SD of 3 biological and 3 technical repeats. Red dashed line indicates baseline permeability of fresh (nonfrozen) cells.

The recovery in TEER and ALP activity post-thaw, when using induced ice nucleation, indicates the presence of a differentiated Caco-2 and HT29-MTX epithelial barrier containing tight junctions and critical biochemical processes. TEER reflects the ionic conductance of the paracellular pathway, whereas the flux of nonelectrolyte tracers can offer insights into paracellular water flow and tight junction pore size.^34^ LY is a small hydrophilic compound that crosses the epithelial barrier mainly via the paracellular space; therefore, it is considered a good marker for tight junctions. LY was applied above the cells (apical portion), and a sample was collected from below the transwell (basal portion) following 1 h of incubation to monitor permeability via fluorescence (Figure 4). As Caco-2 and HT29-MTX were (co)cultured, the formation of a confluent layer with tight junctions decreased the percentage permeability of LY over time (Figure 4B). Expected LY permeability levels were observed after 14 days of culture. HT29-MTX (goblet cells) were introduced into the epithelial barriers to provide mucus secretory cells. Although this introduced advanced intestinal functionality, increased paracellular permeability has been reported,^45^ so the increase in LY permeability compared to Caco-2 cells was expected.

The Caco-2 and/or HT29-MTX monolayers were cryopreserved with 10% DMSO or 10% DMSO and the ice nucleator on day 14, thawed, and assessed for LY permeability over time (Figure 4C–E). For each epithelial model, cryopreservation with the nucleator reduced the increase in immediate LY permeability and ensured that within 7 days, LY permeability was comparable to prefreezing levels. An apparent permeability of ∼5% LY was achieved in just 7 days post-thaw, which is within the range that successfully confirms the presence of tight junctions and normal paracellular diffusion behavior.^46,47^ This was achieved in just 7 days post-thaw, compared to 40 days when thawing from suspension cryopreservation. LY permeability in HT29-MTX and cocultured models was also restored 7 days post-thaw to prefreeze levels when cryopreserved with 10% DMSO and the ice nucleator. In contrast, LY permeability levels were above 50% for all models cryopreserved with 10% DMSO alone even after 7 days post-thaw.

**Figure 5:**
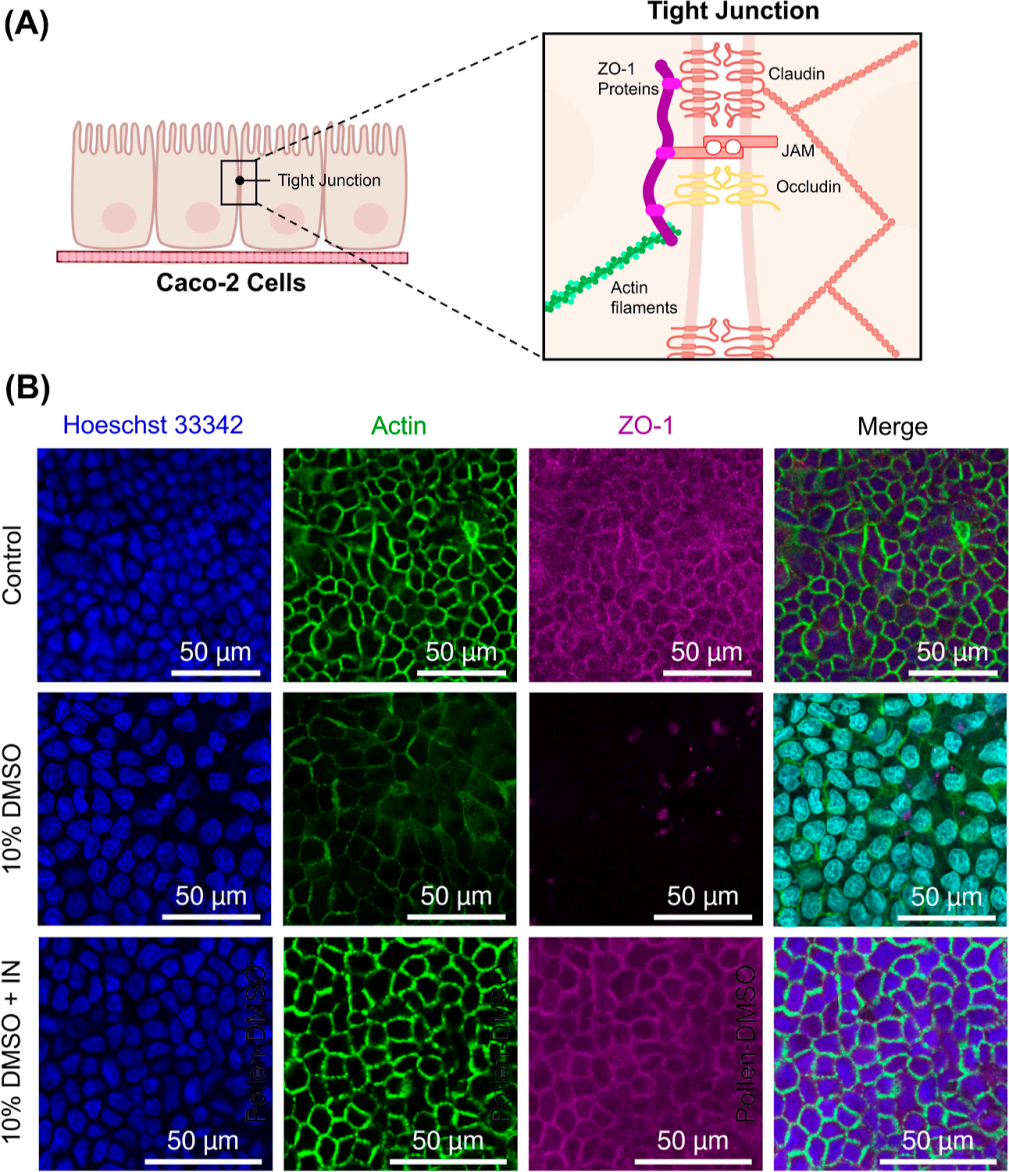
Staining tight junction protein ZO-1. (A) Schematic of the proteins involved in the formation of Caco-2 tight junctions. (B) Confocal images of Caco-2 cells stained for nucleus (Hoeschst 33342, blue), actin (green), and ZO-1 proteins (magenta) before freezing (control) and 24 h after freeze/thaw with 10% DMSO or 10% DMSO plus IN. Scale bar = 50 μm.

To further investigate the protective effect of induced ice nucleation on cryopreserved Caco-2 transwell-cultured cells, confocal microscopy was employed to visualize ZO-1 proteins, a critical component of tight junctions (see Figure 5). Occludin and claudin are bound to the cytoskeleton via scaffolding proteins such as ZO-1, and this assembly plays a pivotal role in the regulation of barrier function,^48^Figure 5A. Thus, actin staining was also used to visualize cytoskeletal integrity before and after cryopreservation with and without ice nucleation after 21 days of culture. In the nonfrozen Caco-2 transwell sample, ZO-1 proteins and actin staining clearly colocalized, indicating the expression of adhesion proteins at tight junction sites and the successful formation of a polarized intestinal epithelial model. Following cryopreservation with ice nucleation, the ZO-1 proteins were preserved at the cellular interface, and cytoskeletal integrity was maintained even 24 h post-thaw. Actin polymerization and cytoskeletal structure provides mechanical support and influences cell migration processes (e.g., vesicle trafficking and cell division). Crucially, actin is connected to transmembrane integrins that bind with collagen, the ECM (extracellular matrix) used to coat the transwells in this study, via several proteins.^49^ Thus, actin preservation during cryopreservation ensures that critical cell–substrate adhesion can be maintained. In comparison, a near-total loss of ZO-1 proteins was observed post-thaw following cryopreservation with 10% DMSO alone, and actin staining was significantly reduced, indicative of cytoskeletal damage. Similar results were obtained for the preservation of Caco-2/HT29-MXT cocultured on transwells shown in Figure S7. The accelerated post-thaw recovery of Caco-2 and Caco-2/HT29-MXT epithelial barriers seen using TEER measurements and ALP and LY assays is likely due to the preservation of crucial tight junction proteins and scaffolds that are bound to the cytoskeleton and, thus, the preservation of cytoskeletal structure. These results are consistent with our hypothesis that reducing water supercooling, using controlled ice nucleation techniques, minimizes cell loss from intracellular ice formation and the disruption of crucial cell–cell junctions required in Caco-2 intestinal models.

**Figure 6:**
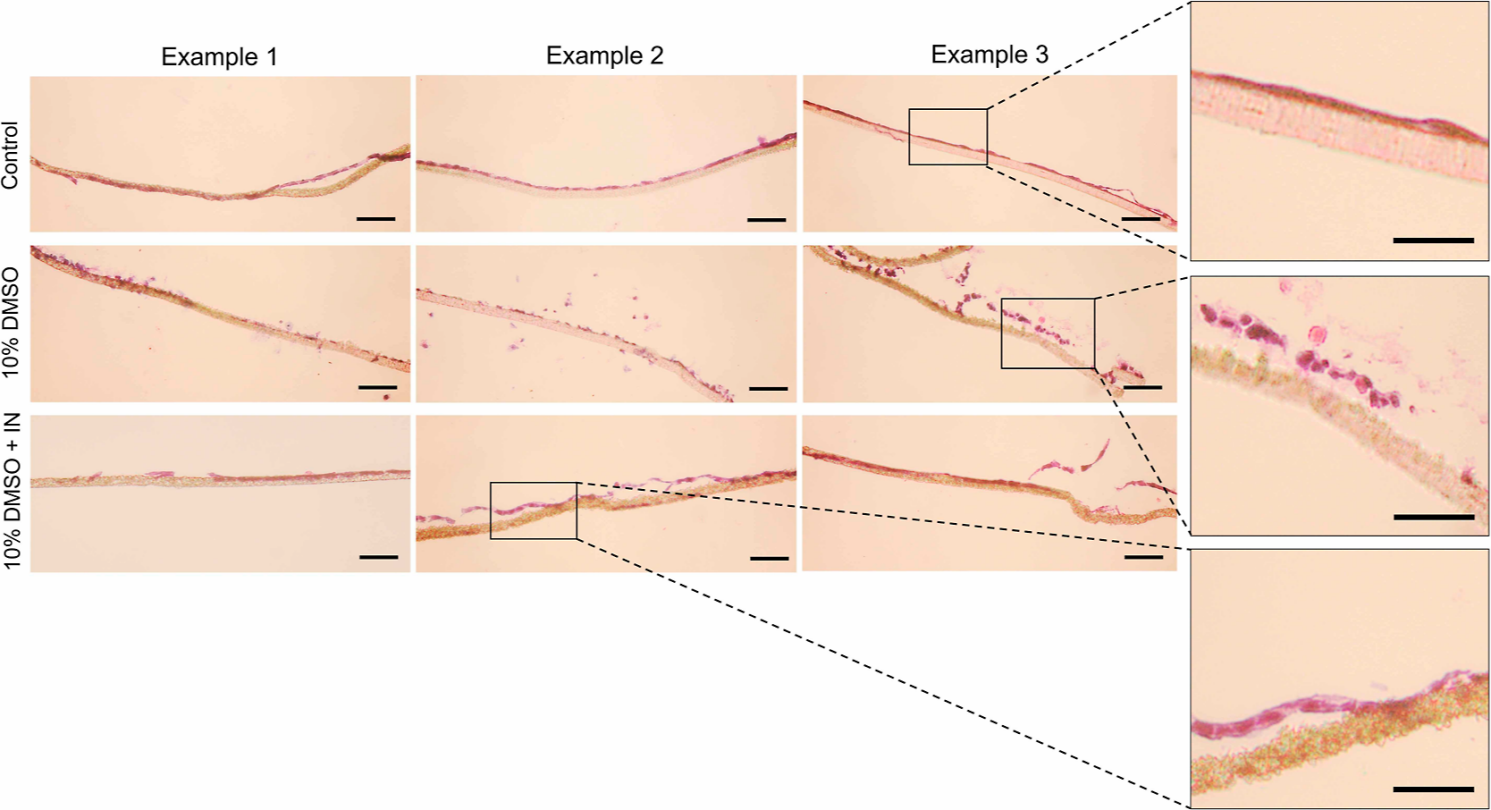
H&E staining of porous membrane sections. Caco-2 cells, cultured on a transwell for 21 days, were sectioned and stained with H&E before and 24 h after freeze/thaw using 10% DMSO and 10% DMSO plus IN to visualize the integrity of the cell monolayer. Scale bar = 100 μm.

To complement the confocal imaging, the Caco-2 epithelial layers (on the transwells) were cryosectioned and stained with H&E to visualize cell monolayer attachment to the transwell 24 h post-thaw, along with possible gaps in cell–cell junctions, Figure 6. After 21 days of culture, a confluent Caco-2 cell monolayer was obtained and attached to the surface of the transwell porous membrane, as expected. Caco-2 cells cryopreserved with 10% DMSO revealed extensive disruption to the cell monolayer network, with a significant loss in tight junctions and the quantity of cells attached to the transwell. Conversely, epithelial layers cryopreserved with ice nucleation displayed a complete monolayer, with tight cell–cell junctions, localized to the porous membrane; providing further evidence that cell–cell contacts are retained when water supercooling is prevented. Along with minimizing disruption of cell–cell junctions during cryopreservation, inducing ice nucleation at warmer temperatures also prevented cell–substrate dissociation.^27,32^

**Figure 7:**
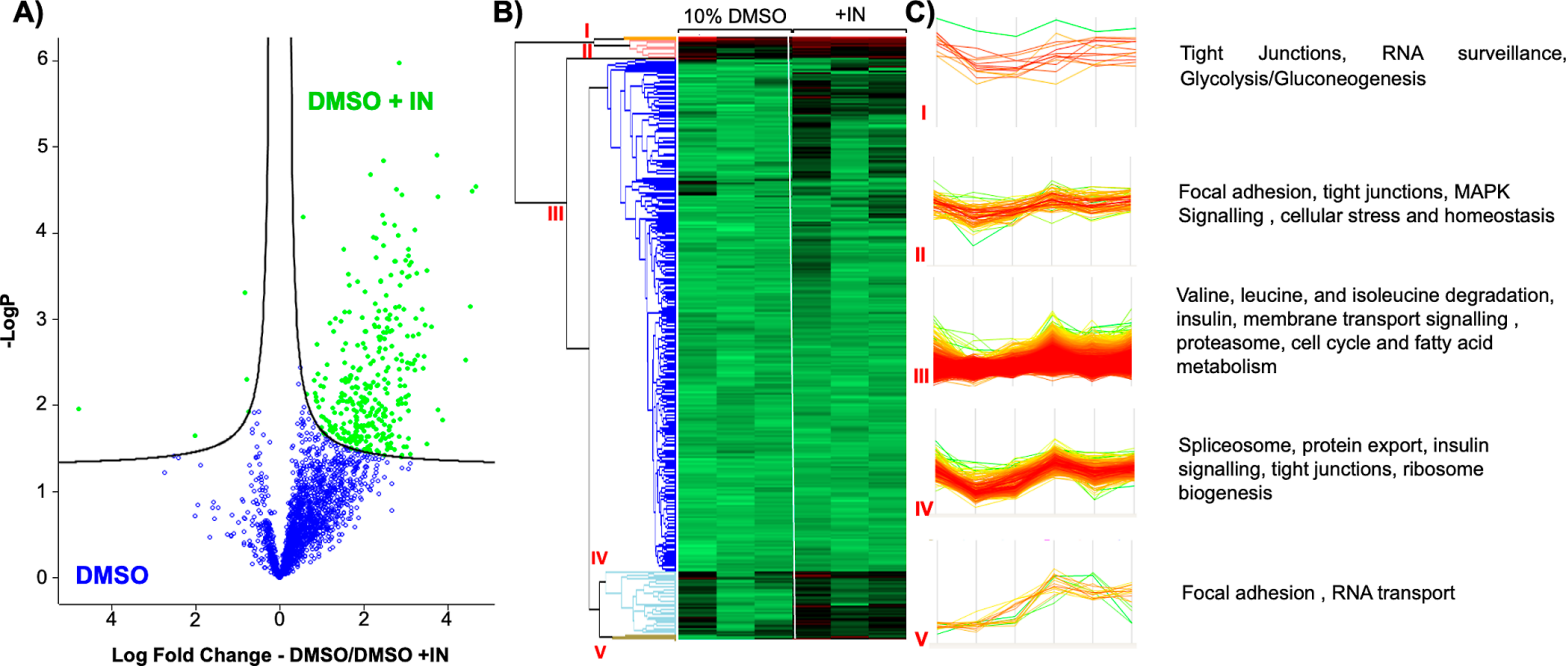
Post-thaw proteomics comparison. Caco-2 cells, cultured on a transwell for 21 days, were cryopreserved with 10% DMSO or 10% DMSO + IN. Proteomics analysis was used to determine protein expression differences post-thaw to identify potential mechanisms of cryo-injury mitigation. (A) Volcano plot; (B) heat map of the expression profile; (C) profile plot derived from heat map from five selected clusters showing significant increase in various pathways, namely, tight junction, MAPK signaling, cellular stress, cell cycle, fatty acid metabolism, RNA surveillance, and RNA transport pathways.

Given the critical requirement of tight junction proteins in establishing epithelial barriers, the protein expression levels of Caco-2 cells cryopreserved with induced ice nucleation were compared against 10% DMSO alone (24 h post-thaw), using whole-cell proteomics, to evaluate the biochemical changes/benefits promoted by ice nucleation, Figure 7. A total of 2315 proteins were successfully identified and quantified, and the robustness of our data is underscored by a Pearson correlation coefficient exceeding 0.99 across all samples. In the volcano plots, 317 upregulated proteins were identified, shown in green, Figure 7A. Several key classes were identified in Figure 7B, which are summarized in Figure 7C and commented upon in the Supporting Information; a complete list of these proteins is provided in Table S1. Crucially, upregulation of claudins, occludins, and ZO proteins were observed. Claudins and occludins are major transmembrane proteins in tight junctions, critical for forming cell barriers, regulating paracellular permeability, and maintaining tight junction integrity.^50^ ZO proteins are cytoplasmic peripheral proteins associated with tight junctions that anchor claudins and occludins to the actin cytoskeleton, regulating tight junction function. The upregulation of these proteins in samples cryopreserved with induced ice nucleation compared to DMSO alone confirms that critical components required in tight junction formation and function are preserved. A range of processes involved in catabolism (amino acid degradation), glucose metabolism, protein turnover, gene expression regulation, and cell division were also significantly higher, confirming that better control over nucleation temperature, to avoid extensive supercooling, helps to promote recovery and protect post-thaw function of many cellular processes.

This is the first comprehensive report on a useful strategy for storing transwell cell monolayers. Soluble ice nucleators, which prevent extensive supercooling of solutions, effectively enabled the cryopreservation of complex transwell-immobilized cell-based models of epithelial tissue. Ice nucleation reduced deleterious intracellular ice formation to improve the recovery and viability of cells post-thaw. The upregulation of ZO proteins and rescue of actin networks for focal adhesion, compared to non-nucleated samples, confirms the preservation of tight junctions and adherence to the permeable support. This was further supported by TEER measurements, confocal imaging, and H&E of cryosections. Crucially, a LY permeability assay, used as a functional test, confirmed that paracellular diffusion of small molecules was normal within 7 days post-thaw. The cryopreservation of Caco-2 transwell models offers a flexible approach to experimental design, better storage and transport capabilities, and recovery of function within 7 days, rather than requiring 21 days of culture.

## Conclusions

Here, we demonstrate the successful cryopreservation of complex epithelial cell models directly on transwell membranes using chemically induced ice nucleation. Caco-2/HT29-MTX were used as a widely deployed intestinal model for drug absorption testing, which requires more than 20 days of culture from suspension cryopreservation. Standard cryoprotectants were unable to cryopreserve epithelial transwell models due to water supercooling, which led to a reduction in post-thaw cell viability and number, loss of cell–cell contacts, and extensive detachment of cells. The soluble polysaccharide ice nucleator employed was able to preserve tight junctions, maintain cell–substrate adhesion, and reduce loss of viability post-thaw. Our cryopreserved model was functional within 7 days post-thaw and can be stored at −80 °C for on-demand use. Normal paracellular permeability of LY along with confocal imaging of ZO-1 proteins and histology confirmed tight junction preservation. Cytoskeletal damage was minimized, which is a critical component to ensure adhesion to the transwells. Finally, whole-cell proteomics confirmed the upregulation of proteins associated with tight junction formation in the cells cryopreserved with induced nucleation. Overall, this is a conclusive data set showing that chemically induced ice nucleation can effectively prevent a broad range of cryopreservation-induced damage in a complex transwell cell culture model. This strategy will enable, with further refinement, banking of assay-ready transwell cell models, speeding up the user time needed from >21 to just 7 days. The ability to bank and distribute complex cellular models from a centralized facility may also accelerate the uptake of nonanimal models, which are difficult to prepare in-house. Finally, this further validates the need to explore physical (e.g., ice nucleation) processes alongside biochemical processes during cryopreservation and discover new tools and materials to modulate these processes.

## Supporting Information Available

Additional cell recovery data, methods for ice nucleator isolation including SDS PAGE analysis, freezing profiles including nucleation temperature comparison data, comparison of temperature profiles with and without IN, FTIR spectra for water-soluble material from *Carpinus betulus* pollen, WST-1 post-thaw (24 h) data for recovery of cryopreserved Caco2 cells, comparative data on change in cytoskeleton behavior using confocal microscopy, volcano plots illustrating protein expression, and key proteomic changes in cryopreserved Caco-2 cells (PDF)
