# Supplementary material for: Cryopreservation and Rapid Recovery of Differentiated Intestinal Epithelial Barrier Cells at Complex Transwell Interfaces Is Enabled by Chemically Induced Ice Nucleation
Source: ACS Appl Mater Interfaces. 2024 Apr 26;16(18):23027–37. doi: 10.1021/acsami.4c03931 (PMC11082836; doi:10.1021/acsami.4c03931)
Supplement: Supplementary file 1 — am4c03931_si_001.pdf [file am4c03931_si_001.pdf]

Supporting Information

Cryopreservation and Rapid Recovery of Differentiated Intestinal Epithelial Barrier Cells at Complex Transwell Interfaces is Enabled by Chemically-Induced Ice Nucleation

Akalabya Bissoyi,[c,d] Yanan Gao,[a,f] Ruben M. F. Tomás,[e] Nina L. H. Kinney,[a,h] Thomas F. Whale,[a,g] Qiongyu Guo,[f] and Matthew I. Gibson[a,b,c,d*]

a) Department of Chemistry, University of Warwick, Coventry, CV4 7AL, United Kingdom

b) Division of Biomedical Sciences, Warwick Medical School, University of Warwick, Coventry, CV4 7AL, United Kingdom

c) Department of Chemistry, University of Manchester, Oxford Road, Manchester, M13 9PL, UK

d) Manchester Institute of Biotechnology, University of Manchester, 131 Princess Street, Manchester, M1 7DN, UK

e) Cryologyx Ltd, Venture Centre, University of Warwick Science Park, Coventry, CV4 7EZ, UK

f) Department of Biomedical Engineering, Southern University of Science and Technology, Shenzhen, Guangdong 518055, China

g) School of Earth and Environment, University of Leeds, Leeds, LS2 9JT, UK

h) Royal Botanic Gardens Kew, Ardingly, West Sussex, RH17 6TN, UK

Corresponding Author Email, matt.gibson@manchester.ac.uk

Isolation of nucleating agent: European hornbeam (*Carpinus betulus*) pollen (0.8 g) purchased from Pharmallerga® was suspended in 20 mL Milli-Q water at 4 °C overnight and sterile filtered using a 0.22 µm filter. Vivaspin 20 centrifugal concentrator of cutoff of 100kDa and 10 kDa (Merck) was employed to segregate molecules of distinct sizes in a solution extract of pollen water. The initial solution underwent centrifugation at 5000 rpm for approximately 30 minutes, with agitation of the supernatant every 5 minutes. Subsequently, the filtrates were withdrawn from the tube and individually preserved for further experiments.

SDS/PAGE: The resultant solution underwent separation through SDS/PAGE under a voltage of 150V for a duration of 15 minutes. After electrophoresis, the gels were stained with Coomassie blue for 60 min. The gel was destained in methanol/H_2O/acetic acid (25:75:5 v/v) for 3 hours (~ 4 × 30 ml) and an image of the gel was obtained using a Biorad Gel Doc XR (BioRad) shown in Figure S1

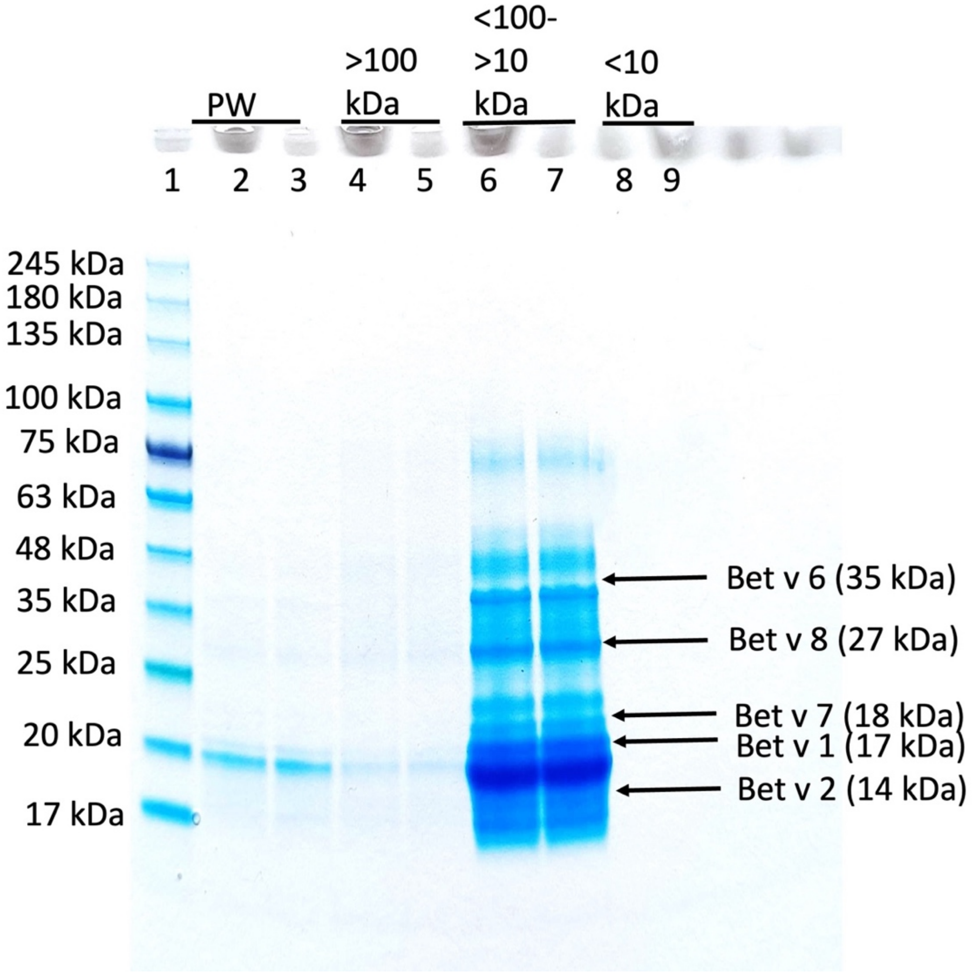

Figure S1: SDS PAGE analysis of before and after the centrifuge fractionation. Lane 1, molecular weight marker. Lane 2 and 3, filtered pollen water solution. Lane 4 and 5, the centrifuge fraction with cutoff of <100 kDa to > 10kDa. Lane 6 and 7, the centrifuge fraction with cutoff of < 10kDa.

The figure shown in supplementary data Figure S1. The centrifuge fraction with cutoff of <100 kDa to > 10kDa exhibits molecular weights (MW) ranging from several to several hundred kDa. The primary birch pollen allergens, namely Bet v6, Bet v8, Bet v7, Bet v1, and Bet v2, are associated with two protein bands of MWs 35 and 27 kDa located in the MMW zone, as well as three proteins of MWs 18, 17, and 14 kDa found in the LMW zone. But in the case of centrifuge fraction < 100kDa fraction has less the primary pollen components.

Ice nucleation temperature determination:

The ice nucleation temperature was determined using two methods, (**1**) a microlitre droplet assay, as described by Whale *et al*,[1] and (**2**) using thermocouples. For method (**1**), approximately 50 one-microlitre droplets were cooled at a rate of 2 °C/min until freezing using a purpose-built aluminium cold stage. The stage was cooled using a TEC1-12704 Peltier thermoelectric cooler connected to a recirculating chiller, driven by a Meerstetter TEC-1091-PT100 Peltier controller. The solution droplets were pipetted onto a 22 mm diameter Hampton Research HR3-231 siliconized glass slide, placed on top of the aluminium cold stage. The stage's temperature was continually monitored to within ± 0.1 °C by two independent Netshushin PT100 platinum resistance thermometers (NR-141-100S-2-1.0-10-2000PLi-A-3) embedded within the cold stage, read by a PicoTech PT-104 Platinum Resistance Data Logger. The droplet nucleation temperatures were determined by the change in droplet opacity visible on freezing, monitored by a camera placed above the cold stage, allowing the fraction of droplets frozen at a given temperature to be determined (figure S2). For method (**2**), Transwell™ inserts were placed in a 24 well plate and the apical portion was filled with either 100 µL of MilliQ water or PWW. T-type thermocouples were placed in the apical chamber of the Transwell™, placed in a -80 °C freezer and the temperature was monitored with a PT100 thermocouple logger integrated into the PicoTech PT-104 was used to monitor the temperature change during cooling (Murray et al. 2023). The ice nucleation temperature was identified as the point where, as the temperature is dropping, a spike in temperature is observed due to the exothermic nature of ice formation (shown in figure S3).

Analysis of Ice Nucleation in microlitre droplets:

Ice nucleation measurements of 1 µl droplets, shown in Fig. S2, clearly indicate the increase in freezing temperature associated with the release of ice nucleating molecules from *Carpinus*

betulus pollen as previously demonstrated[2–4]. The size separation of components of filtered *Carpinus betulus* pollen solution and subsequent ice nucleation measurements indicate that the molecules responsible for ice nucleation at the warmest temperatures are in the largest (>100 kDa) size fraction. This result, and the decreasing nucleation temperatures corresponding to increasingly smaller fractions, is consistent with the concept that larger nucleator surfaces are required to facilitate the formation of critical ice clusters for the nucleation of ice at warmer temperatures, based on classical nucleation theory[5]. The similarity in droplet nucleation temperatures of *Carpinus betulus* pollen solution and the isolated >100 kDa component of this solution demonstrates that the further purification of pollen solutions is possible while still maintaining the action of chemically induced ice nucleation for avoiding intracellular ice crystal formation. This aspect warrants further exploration for future studies using ice nucleators from pollen for biological applications.

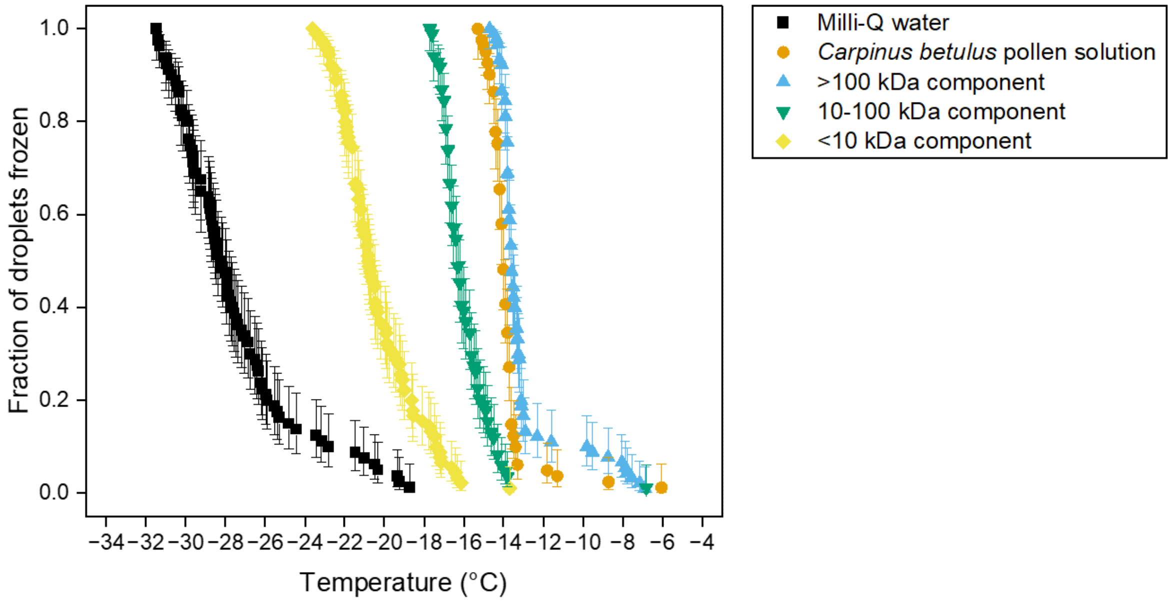

Figure **S2:** Nucleation temperature comparison of 1 µl droplets shown as fraction of droplets frozen against temperature for Milli-Q water (background), *Carpinus betulus* pollen solution and size separated fractions of *Carpinus betulus* pollen solution: >100 kDa, 10-100 kDa and

<10 kDa components.

Analysis of Ice Nucleation in Transwells:

In the following test, we used a transwell to explore nucleation. The findings indicate that, for CPA solutions including pollen water and those without, the nucleation temperature is around -8° C and -10° C, respectively shown in Figure S3.

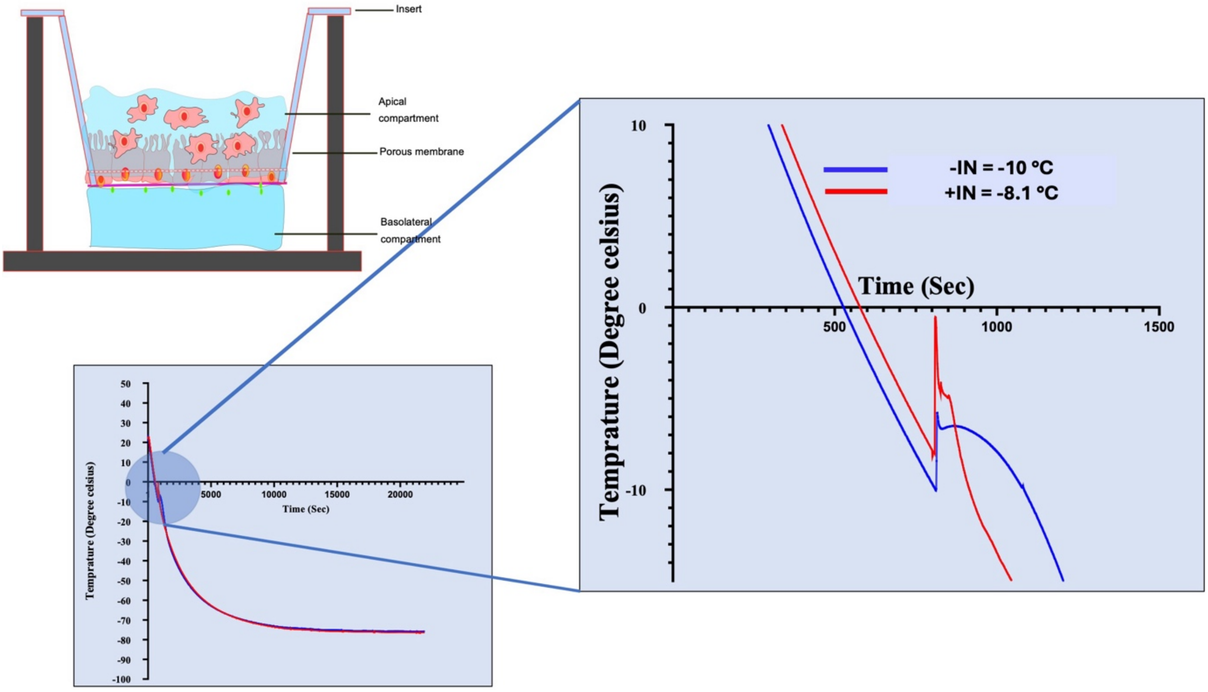

Figure S3: Comparison of temperature profiles recorded with or without IN inside the transwell surface.

FTIR measurements: To determine the infrared absorption spectra for the size-separated components of the *Carpinus betulus* pollen solution, the solutions were freeze-dried, and the infrared absorption spectra of the dry material measured using an Agilent Technologies Cary 630 FTIR Spectrometer.

FTIR results: Figure S4 shows the Fourier-transform infrared (FTIR) spectra of the dried soluble components of these solutions. The absorption spectra for each of the fractions is very similar, with peaks characteristic of polysaccharide absorbances. A slight difference is visible in the fingerprint region at ~1000 cm^{-1} for the most ice nucleation active >100 kDa component.

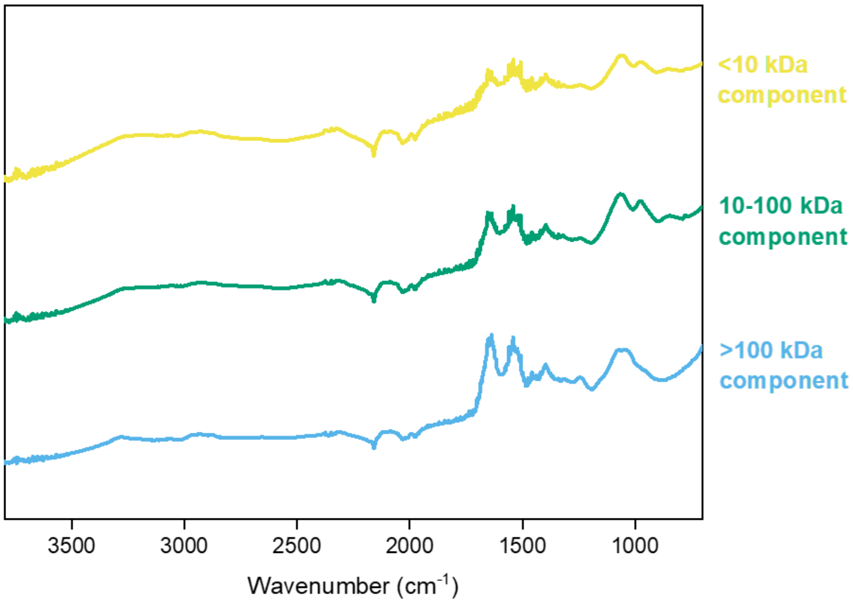

Figure S4: Fourier-transform infrared absorption spectra for water-soluble material from *Carpinus betulus* pollen separated by SDS-PAGE into size fractions: <10 kDa component (low IN activity), 10-100 kDa component (moderate IN activity), and >100 kDa component (high IN activity). The absorption spectra have been normalised and offset along the y-axis.

Post thaw cell viability with different fraction:

To test the effect of different fractions of pollen water solution, we utilised WST-1 proliferation reagent (abcam, Cambridge, UK). The detail procedure is similar to that stated in the material method section. For the experiment, we employed one million Caco2 cells from passage 4. The results show that a 10% DMSO + pollen water solution without centrifuge separation and a fraction greater than 100kDa has a similar recovery rate and even better recovery than 10% DMSO shown in Figure S5.

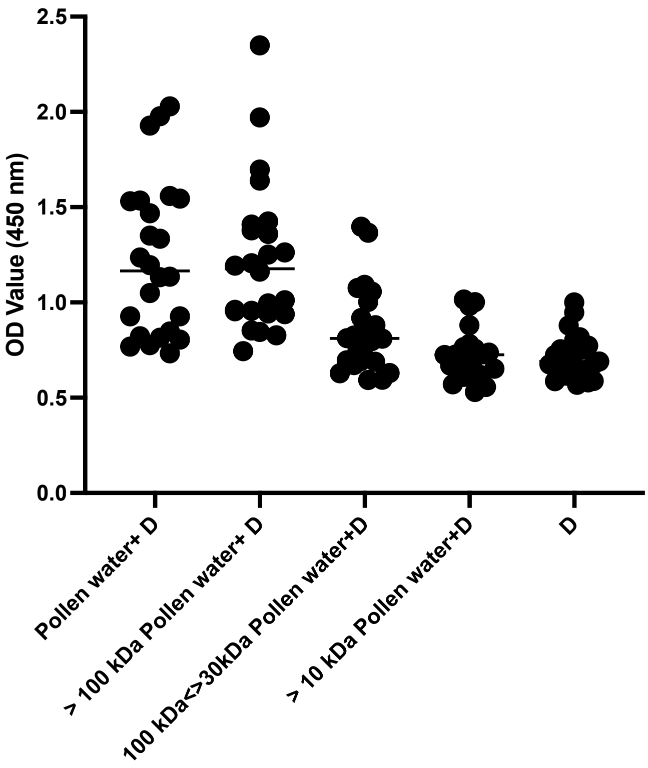

Figure S5**:** WST-1 post-thaw (24 h) recovery of cryopreserved Caco2 cells with various fractions of pollen water solution.

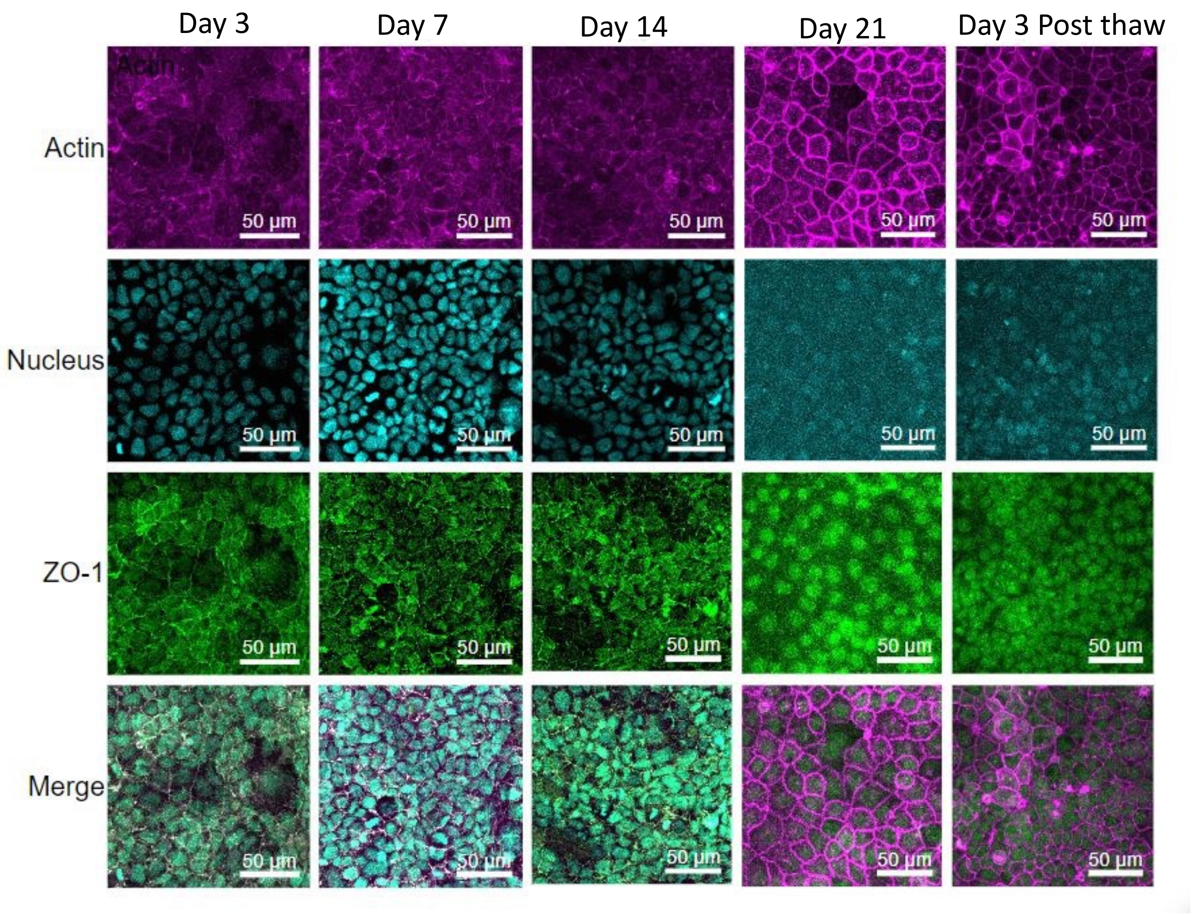

Figure S6: Comparative study on change in cytoskeleton behaviour after post thaw at various time points, the CPA solution used in this study is combination of DMSO-IN. Confocal images of Caco-2 cells stained for Nucleus (Hoeschst 33342, blue), actin (green) and ZO-1 proteins (magenta) before freezing (control) and 24 hours after freeze/thaw with 10% DMSO or 10% DMSO plus IN. Scale bar = 50 µm.

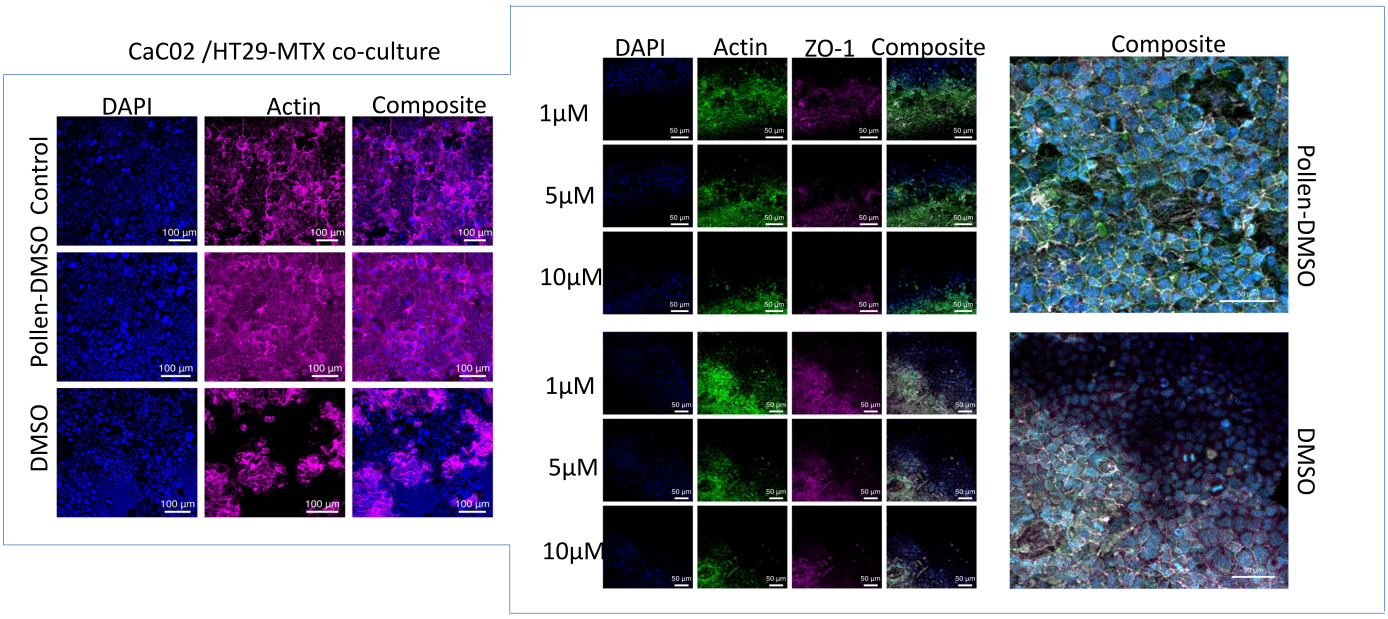

Figure S7: A) Comparative study on change in cytoskeleton behaviour of Caco2/HT29-MTX cocuture system after post thaw, B) Change in expression of actin filament and tight junction protein at various depth in (Hoeschst 33342, blue), actin (green) and ZO-1 proteins (magenta) before freezing (control) and 24 hours after freeze/thaw with 10% DMSO or 10% DMSO plus IN. Scale bar = 50 µm.

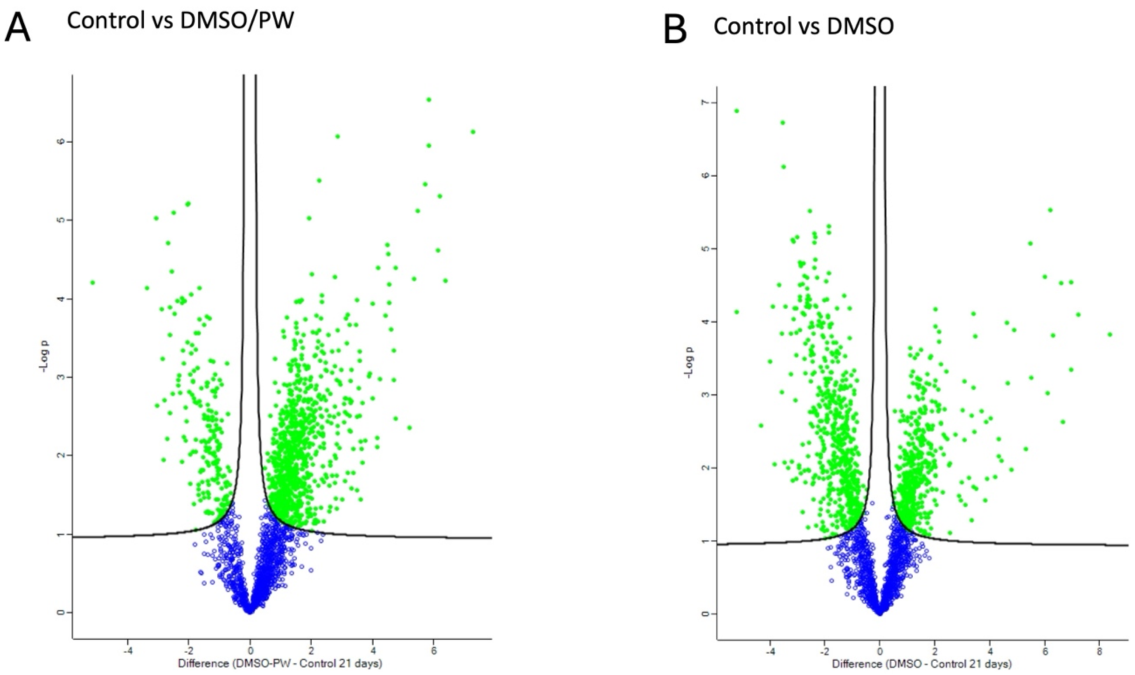

Figure **S8:** Volcano plots illustrating protein expression. (A) Depicts proteins that are differentially expressed between the control group and cryopreserved samples in DMSO, with highlighted (green) markers. (B) Shows proteins with differential expression between the control group and DMSO+IN samples.

Alterations in Metabolic Pathways and Energy Production

The marked upregulation in pathways related to valine, leucine, and isoleucine degradation points to an intensified catabolic activity of these essential amino acids, critical for energy production and metabolite synthesis.[6] Notably, proteins like Shootin-1, Histone deacetylase complex subunit SAP18[7],

and ATP-dependent RNA helicase DDX3X were identified as key players in this process[8]. The proteins are listed in Table S1 in tabular form.

Insulin Signaling and Glucose Metabolism

Our data reveal a pivotal role for Insulin-like growth factor 2 mRNA-binding protein 3 in the insulin signaling pathway, implicating its significance in regulating glucose uptake and metabolism[9]. This finding emphasizes the pathway's importance in maintaining cellular energy homeostasis.

Protein Turnover and Cellular Homeostasis

We observed an upregulation in the proteasome pathway, specifically noting proteins such as the 26S proteasome non-ATPase regulatory subunit 3[10]. This suggests an elevated rate of protein turnover, indicative of a dynamic cellular environment with enhanced protein degradation capabilities.

RNA Dynamics and Gene Expression Regulation

The increased expression of proteins related to RNA transport and degradation highlights their involvement in critical gene expression regulation processes and RNA quality control mechanisms[11].

Impact on Cell Cycle and Apoptosis

Our study also identified significant changes in cell cycle-related proteins, such as Cyclin-dependent kinase 1[12], which may influence cell proliferation. Moreover, the detection of apoptosis-related proteins like Apoptosis-inducing factor 1 points to their involvement in programmed cell death, a key aspect of maintaining tissue homeostasis[13].

Table S1: Key Proteomic Changes in Cryopreserved Caco-2 Cells

**Pathway/Process**	**Upregulated Proteins**	**Downregulated Proteins**	**Implications**
Amino Acid Degradation	Shootin-1, SAP18, DDX3X	-	Enhanced catabolism, energy production
Insulin Signaling	Insulin-like growth factor 2 mRNA-binding protein 3	-	Regulation of glucose metabolism
Proteasome Pathway	26S proteasome non-ATPase regulatory subunit 3	-	Increased protein turnover

RNA Transport/Degradation	Various RNA-related proteins	-	Gene expression regulation
Cell Cycle	Cyclin-dependent kinase 1	-	Influence on cell division
Apoptosis	Apoptosis-inducing factor 1	-	Programmed cell death
Tight Junctions	Claudins, Occludins, ZO Proteins		Critical for maintaining tissue barriers, and these proteins contribute to their structural and functional properties
Lipid Metabolism	-	Apolipoprotein B-100	Altered lipid transport and metabolism
DNA Repair	-	DNA ligase 3	Impaired DNA repair processes
Extracellular Matrix	-	Inter-alpha-trypsin inhibitor heavy chain H4	Changes in tissue structure/function

Reference:

(1) Whale, T. F. Quantification of the Ice Nucleation Activity of Ice-Binding Proteins Using a Microliter Droplet Freezing Experiment. In *Ice Binding Proteins: Methods and Protocols*; Drori, R., Stevens, C., Eds.; Springer US: New York, NY, 2024; pp 121–134. https://doi.org/10.1007/978-1-0716-3503-2_9.

(2) Murray, K. A.; Kinney, N. L. H.; Griffiths, C. A.; Hasan, M.; Gibson, M. I.; Whale, T. F. Pollen Derived Macromolecules Serve as a New Class of Ice-Nucleating Cryoprotectants. *Scientific Reports 2022 12:1* **2022**, *12* (1), 1–11. https://doi.org/10.1038/s41598-022-15545-4.

(3) Gao, Y.; Bissoyi, A.; Kinney, N. L. H.; Whale, T. F.; Guo, Q.; Gibson, M. I. Proline-Conditioning and Chemically-Programmed Ice Nucleation Protects Spheroids during Cryopreservation. *Chemical Communications* **2023**, *59* (59), 9086–9089. https://doi.org/10.1039/D3CC02252H.

(4) Murray, K. A.; Gao, Y.; Griffiths, C. A.; Kinney, N. L. H.; Guo, Q.; Gibson, M. I.; Whale, T. F. Chemically Induced Extracellular Ice Nucleation Reduces Intracellular Ice Formation Enabling 2D and 3D Cellular Cryopreservation. *JACS Au* **2023**, *3* (5), 1314–1320. https://doi.org/10.1021/jacsau.3c00056.

(5) Pummer, B. G.; Budke, C.; Augustin-Bauditz, S.; Niedermeier, D.; Felgitsch, L.; Kampf, C. J.; Huber, R. G.; Liedl, K. R.; Loerting, T.; Moschen, T.; Schauperl, M.; Tollinger, M.; Morris, C. E.; Wex, H.; Grothe, H.; Pöschl, U.; Koop, T.; Fröhlich-Nowoisky, J. Ice Nucleation by Water-Soluble Macromolecules. *Atmos Chem Phys* **2015**, *15* (8), 4077–4091. https://doi.org/10.5194/acp-15-4077-2015.

(6) Dimou, A.; Tsimihodimos, V.; Bairaktari, E. The Critical Role of the Branched Chain Amino Acids (BCAAs) Catabolism-Regulating Enzymes, Branched-Chain Aminotransferase (BCAT)

and Branched-Chain α-Keto Acid Dehydrogenase (BCKD), in Human Pathophysiology. *Int J Mol Sci* **2022**, *23* (7). https://doi.org/10.3390/IJMS23074022.

(7) Zhang, Y.; Iratni, R.; Erdjument-Bromage, H.; Tempst, P.; Reinberg, D. Histone Deacetylases and SAP18, a Novel Polypeptide, Are Components of a Human Sin3 Complex. *Cell* **1997**, *89* (3), 357–364. https://doi.org/10.1016/S0092-8674(00)80216-0.

(8) Soto-Rifo, R.; Ohlmann, T. The Role of the DEAD-Box RNA Helicase DDX3 in MRNA Metabolism. *Wiley Interdiscip Rev RNA* **2013**, *4* (4), 369–385. https://doi.org/10.1002/WRNA.1165.

(9) Kasprzak, A.; Adamek, A. Insulin-Like Growth Factor 2 (IGF2) Signaling in Colorectal Cancer—From Basic Research to Potential Clinical Applications. *International Journal of Molecular Sciences 2019, Vol. 20, Page 4915* **2019**, *20* (19), 4915. https://doi.org/10.3390/IJMS20194915.

(10) Rubio, A. J.; Bencomo-Alvarez, A. E.; Young, J. E.; Velazquez, V. V.; Lara, J. J.; Gonzalez, M. A.; Eiring, A. M. 26S Proteasome Non-ATPase Regulatory Subunits 1 (PSMD1) and 3 (PSMD3) as Putative Targets for Cancer Prognosis and Therapy. *Cells* **2021**, *10* (9). https://doi.org/10.3390/CELLS10092390.

(11) Mittal, N.; Roy, N.; Madan Babu, M.; Chandra Janga, S. *Dissecting the Expression Dynamics of RNA-Binding Proteins in Posttranscriptional Regulatory Networks*. www.pnas.org/cgi/content/full/.

(12) Kalous, J.; Jansová, D.; Šušor, A. Role of Cyclin-Dependent Kinase 1 in Translational Regulation in the M-Phase. *Cells* **2020**, *9* (7). https://doi.org/10.3390/CELLS9071568.

(13) Sevrioukova, I. F. Apoptosis-Inducing Factor: Structure, Function, and Redox Regulation. *Antioxid Redox Signal* **2011**, *14* (12), 2545. https://doi.org/10.1089/ARS.2010.3445.
